# Incidence, predictors and outcomes following primary graft dysfunction after cardiac transplantation

**DOI:** 10.3389/ti.2026.16083

**Published:** 2026-06-30

**Authors:** Isabella Lepore, Jonatan Oras, Andreas Wallinder, Maria Tholén, Aldina Pivodic, Göran Dellgren

**Affiliations:** 1 Department Cardiothoracic Surgery, Sahlgrenska University Hospital, University of Gothenburg, Göteborg, Sweden; 2 Anesthesiology and Intensive Care, Sahlgrenska University Hospital, University of Gothenburg, Göteborg, Sweden; 3 Transplant Institute, Sahlgrenska University Hospital, University of Gothenburg, Göteborg, Sweden; 4 Centre for Person-Centered Care (GPCC), Sahlgrenska University Hospital, University of Gothenburg, Göteborg, Sweden; 5 Department of Surgery, Institute of Clinical Sciences, Sahlgrenska Academy, University of Gothenburg, Göteborg, Sweden; 6 APNC Sweden, Mölndal, Sweden

**Keywords:** graft rejection, heart failure, heart transplant rejection, heart transplantation, primary graft dysfunction

## Abstract

Primary graft dysfunction (PGD) is the leading cause of early morbidity and mortality after heart transplantation (HTx). We retrospectively analyzed 830 consecutive HTx performed between 1984 and 2021. After excluding patients <18 years and those with missing data, 667 adult recipients remained. ISHLT PGD criteria were applied and perioperative variables and outcomes were reviewed. PGD occurred in 70 patients (10.5%), including 41 (6.1%) with left ventricular PGD and 29 (4.3%) with right ventricular PGD. Most LV-PGD cases were severe (88%). Patients with PGD were younger and more frequently had pretransplant dialysis, ventricular assist device support, or prior cardiac surgery. No donor-related factors were associated with PGD. Recipient-related factors and longer cardiopulmonary bypass time were associated with increased risk. PGD was associated with prolonged mechanical ventilation and ICU stay, and increased need for mechanical circulatory support, dialysis, reoperation, and treatment for sepsis. Mortality or re-transplantation was significantly higher in PGD patients at 30 days (45% vs. 3%) and 1 year (51% vs. 8%; HR 6.89, 95% CI 4.01–11.83, p < 0.0001).

## Graphical Abstract

**Figure:**
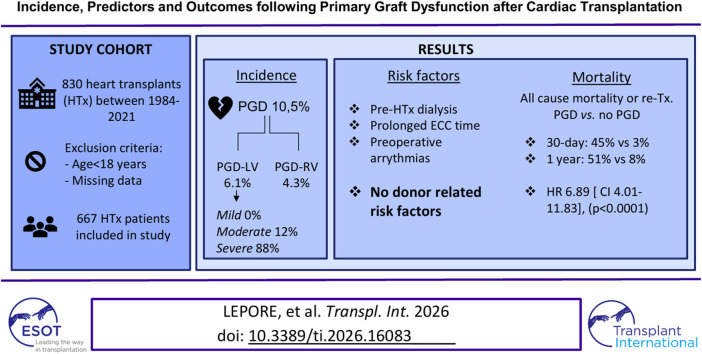

## Introduction

Heart transplantation (HTx) is the gold standard treatment for end-stage heart failure, with close to 9000 patients undergoing the procedure worldwide every year. In the modern era 1-year survival is about 90% and a median-survival of 12.5 years, but even longer survival has been observed in the Scandinavian countries [1–4].

Primary graft dysfunction (PGD) is the leading cause of morbidity and mortality within the first 30 days after HTx [5] and presents as left-, right-, or biventricular allograft failure [3, 6]. Various risk factors for graft dysfunction have been reported, such as graft ischemia time, high donor and recipient age and mechanical circulatory support before transplantation [7–11].

Over time, different definitions of graft dysfunction have been used, leading to challenges in comparing data across time periods and publications. This is exemplified by the reported incidences ranging from 2.5% to 28.2%. In 2014, ISHLT agreed on consensus criteria for graft dysfunction separating primary from secondary graft dysfunction [12]. The latter, a consequence of, for example, acute rejection, severe bleeding or a surgical complication, while primary graft dysfunction has no obvious discernable cause. Since the ISHLT definition was introduced, transplant centers have utilized the criteria to better identify incidence, risk factors, and mortality, providing a more precise assessment compared to evaluations before 2014. In 2020, a meta-analysis by Buchan et al. reported a pooled incidence with moderate certainty ranging from 1.6% to 7.7%, depending on the subtype of PGD [7]. Further research is essential to achieve a comprehensive understanding of this condition and therefore we investigated our experience of PGD. The aim of this study was to investigate the incidence of PGD thoroughly checked from source data according to the ISHLT guidelines at our center, to identify predictors for PGD, and to study the impact of PGD on the short-term outcome.

## Patients and methods

### Patient cohort

The study was approved by the Swedish National Research Ethics Committee, following the Helsinki Declaration (Diary number 728-12, amendment 2020-04281). Being a retrospective study, patient consent was not deemed necessary and waived by the ethical committee. Inclusion criteria were all patients >18 years having had a HTx procedure between June 1984 and December 2021 at Sahlgrenska University Hospital, Sweden. Patients with incomplete medical records preventing assessment of early graft function were excluded. For further details regarding study cohort, see Supplementary Figure SA1.

### PGD definitions

Primary graft dysfunction (PGD) is defined in the ISHLT consensus document as acute onset of heart failure affecting the right, left, or both ventricles within the first 24 h after HTx. Additionally, to be classified as PGD, the graft dysfunction may not be attributed to other causes, such as uncontrolled bleeding, surgical complications, pulmonary hypertension or acute rejection. In these circumstances the graft dysfunction is labeled secondary. PGD is categorized into two entities: PGD-Right ventricle (PGD-RV) and PGD-Left ventricle (PGD-LV). The subclassification of PGD-LV includes mild, moderate, and severe, providing a detailed spectrum of severity. For a more comprehensive understanding, see Supplementary Figure SA1.

### Data collection and variables

**FIGURE 1:**
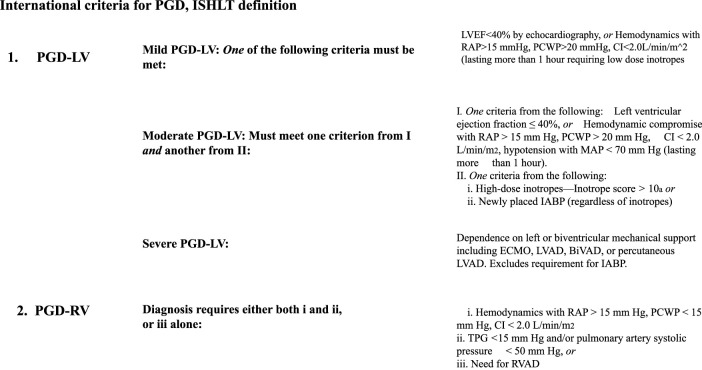
Definition for primary graft dysfunction adopted from ISHLT consensus report. PGD, primary graft dysfunction. LV, left ventricle; RV, right ventricle; RAP, right atrial pressure; PCWP, pulmonary capillary wedge pressure; MAP, mean arterial pressure; CI, cardiac index; ECMO, extracorporeal membrane oxygenation; IABP, intra-aortic balloon pump; LVAD, left ventricular assist device; BiVAD, biventricular assist device; RVAD, right ventricular assist device; TPG, transpulmonary gradient.

Data was collected retrospectively for all patients included in the study. We are maintaining an updated database of the HTx patients including main patient characteristics as well as follow-up data at our center. However, specific information on PGD has not continuously been collected. Therefore, all anesthesia records and intensive care unit (ICU) charts were retrieved and analyzed (including use of inotropic agents). ICU charts records included hourly measurements of central venous pressure (CVP), continuous cardiac output (CCO), mean arterial pressure (MAP). Pulmonary capillary wedge pressure (PCWP) and mean pulmonary arterial pressure (MPAP) via a Swan-Ganz were recorded at regular intervals. From CCO the cardiac index (CI) was calculated using the preoperative body surface area of the patient. Trans pulmonary gradient (TPG) was calculated under the assumption that PCWP is equal to the left atrial pressure (LAP) and was deducted from MPAP to receive the TPG. All hemodynamic parameters must have met the criteria in Figure 1 for at least >1 h for the diagnosis of PGD to be set. To determine the inotropic score, the highest dosage using the concentration in µg/kg/min per inotrope during the first 24 h was selected [12].

By analyzing all the perioperative and postoperative data during the first 24 h in the ICU we were able to identify a graft dysfunction (GD) and non-GD cohort. The data was meticulously reviewed to be able to determine whether PGD had occurred. The operative notes as well as the anesthesia reports were analyzed to exclude secondary graft dysfunction. The PGD group was then divided into four sub-groups using the ISHLT definition (Supplementary Figure SA1).

### Statistics

Continuous variables are presented by mean, standard deviation, median, minimum and maximum, and categorical variables by frequency and percentage.

For tests between PGD and no PGD group Fisher’s exact test was used for dichotomous variables, Mantel-Haenszel Chi-square trend test for ordered categorical variables, Chi-square test for non-ordered categorical variables and Mann-Whitney U-test for continuous variables. In the analyses on HTx level the observations were assumed to be independent given the small number of hearts with previous re-HTx (n = 21). All completed transplants were divided into three time-era periods (1984–1999, 2000–2010 and 2011-2022). Associations between pre-HTx and intraoperative clinically relevant a-priori selected variables and incidence of PGD were investigated using logistic regression, adjusted for age and sex. A multivariable model was obtained by running a stepwise selection (forward and backward) and keeping only statistically significant variables beside adjustment for age and sex. From these analyses odds-ratios with 95% confidence intervals (CI), p-values, and area under receiver operating characteristic curve (AUC or c-statistics) were presented. In these analyses only the first HTx was studied. Results from stepwise models should be interpreted as exploratory. No evidence of significant collinearity among the included variables was observed. Missing data were limited and managed using a complete-case approach in the multivariable models, with negligible expected impact on the results.

Associations between pre-HTx and intraoperative variables and the primary outcome all-cause death or re-HTx during first year were performed using Cox regression, adjusted for age and sex. A multivariable model was obtained by running a stepwise selection (forward and backward) and keeping only statistically significant variables beside adjustment for age and sex. From these analyses hazard ratios (HR) and adjusted HR (aHR) with 95% CI and p-values were presented. In the selected multivariable model, the assumption of proportional hazards was tested by introducing interaction terms between variables and log(time) which were non-significant. Hence, proportional hazards could be assumed. Descriptively, survival data was presented by Kaplan-Meier plots. In these analyses only first HTx was studied.

All tests were two-tailed and conducted at 0.05 significance level. All analyses were performed using SAS software version 9.4 (SAS Institute Inc., Cary, NC, USA).

## Results

### Patient characteristics

Among 830 subjects that underwent HTx between June 1984 and December 2021, 103 (12.4%) were excluded due to age <18 years and 60 (7.2%) due to incomplete medical records. A total of 667 (80.4%) HTx, in 646 unique patients, met the inclusion criteria and were included in the study (Supplementary Figure SA1). Of these, 504 (75.6%) were men and 163 (24.4%) women.

### Incidence of PGD

In total, 16 patients had secondary graft dysfunction due to, e.g., uncontrolled bleeding, stenosis of the vena cava anastomoses or hyperacute rejection. Our primary endpoint PGD was evident in 70 HTx patients and the overall incidence of PGD was 10.5%. This group was now compared to the non-PGD cohort that consisted of 597 HTx in 580 unique patients.

Other secondary endpoints, such as PGD-LV (n = 41,58.6%)) was more common than PGD-RV (n = 29) of the PGD cases. Among patients with PGD-LV, 5 had (12%) moderate and 36 (88%) the severe form. Patients who developed PGD were on average younger than those in the non-PGD group (45.5 vs. 49.7, p = 0.03). They were also shorter and had a lower body weight (173 cm vs. 176 cm; p = 0.0063 and 75 kg vs. 80kg; p = 0.0067, respectively). Patients with and without PGD had different diagnoses leading to HTx (p = 0.02). The PGD group more frequently had dialysis preoperatively (7.7% vs. 1.7%; p = 0.012), a history of cardiac surgery (60.3% vs. 37.3%, p = 0.0004) and pre-HTx ventricular assist device (VAD) (32.8% vs. 18.8%, p = 0.0098) compared to the non-PGD group. For further details see Table 1.

**TABLE 1:** Preoperative patient characteristics.

Variable	Total N = 667	No PGD N = 597	PGD N = 70	No PGD vs. PGD p-value	PGD-LV N = 41	PGD-RV N = 29
Sex	​	​	​	1.00	​	​
Male	504 (75.6%)	451 (75.5%)	53 (75.7%)	​	33 (80.5%)	20 (69.0%)
Female	163 (24.4%)	146 (24.5%)	17 (24.3%)	​	8 (19.5%)	9 (31.0%)
Age	49.3 ± 12.9 52.6 (18.0–70.5) n = 667	49.7 ± 12.6 52.8 (18.0–70.5) n = 597	45.5 ± 14.5 49.9 (18.2–67.4) n = 70	0.030	45.1 ± 14.4 49.8 (18.4–67.4) n = 41	46.1 ± 14.8 50.1 (18.2–65.5) n = 29
Age category	​	​	​	0.0089	​	​
<30y	75 (11.2%)	59 (9.9%)	16 (22.9%)	​	10 (24.4%)	6 (20.7%)
30-<40y	79 (11.8%)	73 (12.2%)	6 (8.6%)	​	4 (9.8%)	2 (6.9%)
40-<50y	138 (20.7%)	125 (20.9%)	13 (18.6%)	​	7 (17.1%)	6 (20.7%)
50-<60y	235 (35.2%)	207 (34.7%)	28 (40.0%)	​	16 (39.0%)	12 (41.4%)
60 + y	140 (21.0%)	133 (22.3%)	7 (10.0%)	​	4 (9.8%)	3 (10.3%)
Weight (kg)	79.1 ± 16.2 79.0 (26.0–129.0) n = 662	79.7 ± 16.2 80.0 (26.0–129.0) n = 593	74.5 ± 15.6 73.0 (42.0–111.0) n = 69	0.0067	74.5 ± 14.6 71.0 (50.0–110.0) n = 40	74.6 ± 17.2 75.0 (42.0–111.0) n = 29
Length (cm)	175.6 ± 9.2 176 (139–205) n = 662	176.0 ± 9.1 176 (139–205) n = 593	172.8 ± 8.9 173 (152–190) n = 69	0.0063	173.1 ± 8.9 173 (154–189) n = 40	172.3 ± 9.0 172 (152–190) n = 29
Body mass index (kg/m^2)	25.5 ± 4.3 25.2 (13.5–40.7) n = 661	25.6 ± 4.3 25.2 (13.5–40.7) n = 592	24.9 ± 4.4 24.5 (15.5–34.1) n = 69	0.22	24.8 ± 4.1 24.5 (16.9–34.1) n = 40	25.0 ± 4.9 24.8 (15.5–33.8) n = 29
Diagnosis	​	​	​	0.020	​	​
Ischemic cardiomyopathy	158 (24.5%)	138 (23.9%)	20 (29.4%)	​	10 (25.0%)	10 (35.7%)
Dilated cardiomyopathy	334 (51.7%)	311 (53.8%)	23 (33.8%)	​	14 (35.0%)	9 (32.1%)
Myocarditis	12 (1.9%)	10 (1.7%)	2 (2.9%)	​	1 (2.5%)	1 (3.6%)
Re-transplantation	21 (3.3%)	17 (2.9%)	4 (5.9%)	​	3 (7.5%)	1 (3.6%)
Valve disease	12 (1.9%)	11 (1.9%)	1 (1.5%)	​	1 (2.5%)	0 (0.0%)
Congenital heart disease	43 (6.7%)	33 (5.7%)	10 (14.7%)	​	5 (12.5%)	5 (17.9%)
Arrhythmogenic right valve dysplasia	17 (2.6%)	16 (2.8%)	1 (1.5%)	​	1 (2.5%)	0 (0.0%)
Restrictive cardiomyopathy	14 (2.2%)	10 (1.7%)	4 (5.9%)	​	3 (7.5%)	1 (3.6%)
Hypertrophic cardiomyopathy	27 (4.2%)	24 (4.2%)	3 (4.4%)	​	2 (5.0%)	1 (3.6%)
Amyloidosis	7 (1.1%)	7 (1.2%)	0 (0.0%)	​	​	​
Restrictive pericarditis	1 (0.2%)	1 (0.2%)	0 (0.0%)	​	​	​
Missing	21	19	2	​	1	1
Smoking	​	​	​	0.34	​	​
No	363 (55.8%)	320 (54.9%)	43 (64.2%)	​	25 (64.1%)	18 (64.3%)
Smoked within 6 months	52 (8.0%)	48 (8.2%)	4 (6.0%)	​	0 (0.0%)	4 (14.3%)
Previous smoker (>6 months)	235 (36.2%)	215 (36.9%)	20 (29.9%)	​	14 (35.9%)	6 (21.4%)
Missing	17	14	3	​	2	1
Dialysis	​	​	​	0.012	​	​
No	630 (97.7%)	570 (98.3%)	60 (92.3%)	​	34 (91.9%)	26 (92.9%)
Yes	15 (2.3%)	10 (1.7%)	5 (7.7%)	​	3 (8.1%)	2 (7.1%)
Missing	22	17	5	​	4	1
Previous sternotomy	​	​	​	0.0004	​	​
No	397 (60.3%)	370 (62.7%)	27 (39.7%)	​	16 (40.0%)	11 (39.3%)
Yes	261 (39.7%)	220 (37.3%)	41 (60.3%)	​	24 (60.0%)	17 (60.7%)
Missing	9	7	2	​	1	1
Previous heart transplantation	​	​	​	0.26	​	​
No	646 (96.9%)	580 (97.2%)	66 (94.3%)	​	38 (92.7%)	28 (96.6%)
Yes	21 (3.1%)	17 (2.8%)	4 (5.7%)	​	3 (7.3%)	1 (3.4%)
Amiodarone	​	​	​	0.20	​	​
No	511 (79.0%)	464 (79.7%)	47 (72.3%)	​	27 (71.1%)	20 (74.1%)
Yes	136 (21.0%)	118 (20.3%)	18 (27.7%)	​	11 (28.9%)	7 (25.9%)
Missing	20	15	5	​	3	2
Inotrope	​	​	​	0.17	​	​
No	478 (74.8%)	435 (75.7%)	43 (67.2%)	​	25 (69.4%)	18 (64.3%)
Yes	161 (25.2%)	140 (24.3%)	21 (32.8%)	​	11 (30.6%)	10 (35.7%)
Missing	28	22	6	​	5	1
Missing	14	9	5	​	3	2
NYHA	​	​	​	0.37	​	​
I-II	32 (5.0%)	31 (5.4%)	1 (1.6%)	​	1 (2.7%)	0 (0.0%)
III	434 (68.5%)	390 (68.3%)	44 (69.8%)	​	25 (67.6%)	19 (73.1%)
IV	168 (26.5%)	150 (26.3%)	18 (28.6%)	​	11 (29.7%)	7 (26.9%)
Missing	33	26	7	​	4	3
Arrhythmias	​	​	​	0.039	​	​
No	425 (65.7%)	390 (67.0%)	35 (53.8%)	​	18 (48.6%)	17 (60.7%)
Yes	222 (34.3%)	192 (33.0%)	30 (46.2%)	​	19 (51.4%)	11 (39.3%)
Missing	20	15	5	​	4	1
VAD	​	​	​	0.0098	​	​
No	521 (79.8%)	476 (81.2%)	45 (67.2%)	​	25 (64.1%)	20 (71.4%)
Yes	132 (20.2%)	110 (18.8%)	22 (32.8%)	​	14 (35.9%)	8 (28.6%)
Missing	14	11	3	​	2	1
Calendar year	​	​	​	0.07	​	​
1984–1999	201 (30.1%)	178 (29.8%)	23 (32.9%)	​	15 (36.6%)	8 (27.6%)
2000–2010	176 (26.4%)	149 (25.0%)	27 (38.6%)	​	14 (34.1%)	13 (44.8%)
2011–2022	290 (43.5%)	270 (45.2%)	20 (28.6%)	​	12 (29.3%)	8 (27.6%)

Data are presented as mean ± standard deviation, median (range) and number of observations, or number (percentage).

For test between two groups with respect to dichotomous variables Fisher’s exact test was used, for non-ordered categorical variables Chi-square test, for ordered categorical variables Mantel-Haenszel Chi-square trend test, and for continuous variables Mann-Whitney U-test.

PGD, primary graft dysfunction; LV, left ventricle; RV, right ventricle; VAD, Ventricular assist device; NYHA, New York Heart Association.

### Donor characteristics

Other secondary outcomes, donor age for the whole cohort was 41.6 ± 14.9 years. In the donor population, men were overrepresented with 431 individuals (64.6%) compared to 236 women (35.4%). Intracerebral hemorrhage emerged as the predominant cause of death (49.0%), followed by trauma (26.7%). There were no differences in donor characteristics between recipients with or without PGD. Donor-related risk factors are presented in Table 2.

**TABLE 2:** Donor characteristics.

Variable	Total N = 667	No PGD N = 597	PGD N = 70	No PGD vs. PGD p-value	PGD-LV N = 41	PGD-RV N = 29
Donor sex	​	​	​	0.60	​	​
Male	431 (64.6%)	388 (65.0%)	43 (61.4%)	​	26 (63.4%)	17 (58.6%)
Female	236 (35.4%)	209 (35.0%)	27 (38.6%)	​	15 (36.6%)	12 (41.4%)
Donor age (years)	41.6 ± 14.9 43.0 (12.0–73.0) n = 666	41.5 ± 15.1 43.0 (12.0–73.0) n = 596	42.9 ± 12.5 46.0 (15.0–65.0) n = 70	0.50	41.7 ± 13.0 44.0 (15.0–65.0) n = 41	44.7 ± 11.9 49.0 (21.0–64.0) n = 29
Donor blood group	​	​	​	0.99	​	​
0	287 (43.2%)	256 (43.1%)	31 (44.3%)	​	20 (48.8%)	11 (37.9%)
A	296 (44.6%)	265 (44.6%)	31 (44.3%)	​	17 (41.5%)	14 (48.3%)
B	72 (10.8%)	65 (10.9%)	7 (10.0%)	​	4 (9.8%)	3 (10.3%)
AB	9 (1.4%)	8 (1.3%)	1 (1.4%)	​	0 (0.0%)	1 (3.4%)
Missing	3	3	0	​	​	​
Donor height (cm)	176.1 ± 10.7 177 (80–205) n = 662	176.1 ± 10.2 176 (89–205) n = 592	175.8 ± 14.5 177 (80–195) n = 70	0.62	177.3 ± 8.3 177 (160–195) n = 41	173.6 ± 20.2 175 (80–195) n = 29
Donor weight (kg)	78.8 ± 16.8 77.0 (45.0–192.0) n = 664	78.6 ± 16.5 77.0 (45.0–192.0) n = 594	81.0 ± 19.0 76.0 (52.0–175.0) n = 70	0.51	80.8 ± 16.8 78.0 (58.0–135.0) n = 41	81.2 ± 22.0 75.0 (52.0–175.0) n = 29
Reason for death	​	​	​	0.36	​	​
Unknown	25 (4.3%)	25 (4.8%)	0 (0.0%)	​	​	​
ICB	282 (49.0%)	249 (48.1%)	33 (56.9%)	​	19 (61.3%)	14 (51.9%)
Trauma	154 (26.7%)	138 (26.6%)	16 (27.6%)	​	9 (29.0%)	7 (25.9%)
Thromboembolic	17 (3.0%)	16 (3.1%)	1 (1.7%)	​	1 (3.2%)	0 (0.0%)
Other	98 (17.0%)	90 (17.4%)	8 (13.8%)	​	2 (6.5%)	6 (22.2%)
Missing	91	79	12	​	10	2

Data are presented as mean ± standard deviation, median (range) and number of observations, or number (percentage).

For test between two groups with respect to dichotomous variables Fisher’s exact test was used, for non-ordered categorical variables Chi-square test, and for continuous variables Mann-Whitney U-test.

PGD, primary graft dysfunction; ICB, intracranial cerebral bleeding; LV, left ventricle; RV, right ventricle.

### Intraoperative findings

A trend towards longer graft ischemic time in patients with PGD was observed (195 min vs. 181 min, p = 0.06) (Table 3). When re-transplantations were excluded from the analysis, this difference was significant (196 min vs. 181 min, p = 0.035). Patients with PGD had longer extra corporeal circulation (ECC) times (median 193.0 min vs. 145.0 min, p-value <0.0001) indicating more complex procedures.

**TABLE 3:** Intraoperative data.

Variable	Total N = 667	No PGD N = 597	PGD N = 70	No PGD vs. PGD p-value	PGD-LV N = 41	PGD-RV N = 29
Ischemic time (min)	182.7 ± 59.1 185.5 (44.0–431.0) n = 642	181.3 ± 59.5 185.0 (44.0–431.0) n = 576	194.7 ± 54.9 196.5 (51.0–375.0) n = 66	0.06	186.8 ± 50.5 191.0 (51.0–284.0) n = 37	204.8 ± 59.5 209.0 (73.0–375.0) n = 29
ECC	159.9 ± 65.6 149.0 (56.0–583.0) n = 653	153.4 ± 56.2 145.0 (56.0–583.0) n = 584	214.6 ± 103.7 193.0 (82.0–495.0) n = 69	<0.0001	225.3 ± 110.6 198.0 (92.0–495.0) n = 40	199.7 ± 93.1 169.0 (82.0–446.0) n = 29
Cross clamp duration at implant	96.7 ± 54.9 80.0 (17.0–450.0) n = 479	96.2 ± 54.9 79.0 (17.0–450.0) n = 428	101.4 ± 55.3 85.0 (23.0–257.0) n = 51	0.62	101.5 ± 52.7 83.0 (43.0–202.0) n = 31	101.3 ± 60.7 95.5 (23.0–257.0) n = 20

Data are presented as mean ± standard deviation, median (range) and number of observations.

For test between two groups Mann-Whitney U-test was used for continuous variables.

PGD, primary graft dysfunction; LV, left ventricle; RV, right ventricle; ECC, extra corporeal circulation.

### Postoperative findings

Postoperative secondary outcome data is presented in Table 4. The number of days in the ICU was longer in PGD patients with an average difference of more than 2 weeks (22.1 ± 23.1 vs. 7.8 ± 12.9, p < 0.0001). Postoperative mechanical circulatory support (MCS) was seen in 75.4% of patients with PGD and in 0.9% in the non-PGD group (p < 0.0001). PGD subjects required re-operation to a greater extent (70.1% vs. 27.2%, p < 0.0001). Post-operative dialysis was more frequent in the PGD cohort (50.0% vs. 19.0%, p < 0.0001) and the time with mechanical ventilation was longer (326 ± 458 h vs. 63 ± 167h, p < 0.0001). The proportion of patients who experienced post-operative sepsis was also higher in the PGD group (28.1% vs. 8.9%, p < 0.0001).

**TABLE 4:** Postoperative data.

Variable	Total N = 646	No PGD N = 580	PGD N = 66	No PGD vs. PGD p-value	PGD-LV N = 38	PGD-RV N = 28
Mechanical ventilation time (h)	88.9 ± 229.7 10 (0–1906) n = 629	61.6 ± 166.3 9 (0–1531) n = 567	338.5 ± 465.1 126 (2–1906) n = 62	<0.0001	306.0 ± 499.3 79 (2–1906) n = 35	380.6 ± 422.3 255 (5–1691) n = 27
Postoperative MCS	​	​	​	<0.0001	​	​
No	576 (91.3%)	561 (99.1%)	15 (23.1%)	​	6 (16.2%)	9 (32.1%)
Yes	55 (8.7%)	5 (0.9%)	50 (76.9%)	​	31 (83.8%)	19 (67.9%)
Missing	15	14	1	​	1	0
ICU time (days)	8.9 ± 14.6 4 (0–219) n = 607	7.7 ± 12.9 4 (0–219) n = 561	23.4 ± 23.6 13 (1–81) n = 46	<0.0001	23.0 ± 26.9 10 (1–81) n = 23	23.9 ± 20.4 20 (3–77) n = 23
In-hospital stay (days)	32.1 ± 17.7 29 (0–153) n = 640	32.0 ± 15.6 29 (0–153) n = 574	33.2 ± 30.9 26 (0–141) n = 66	0.08	26.5 ± 32.0 16 (0–141) n = 38	42.4 ± 27.3 33 (1–100) n = 28
Re-operation at first admission	​	​	​	<0.0001	​	​
No	426 (68.6%)	408 (73.1%)	18 (28.6%)	​	9 (25.0%)	9 (33.3%)
Yes	195 (31.4%)	150 (26.9%)	45 (71.4%)	​	27 (75.0%)	18 (66.7%)
Missing	25	22	3	​	2	1
Inotrope score	39.4 ± 35.1 31.1 (0.0–186.0) n = 615	38.3 ± 34.5 29.0 (0.0–186.0) n = 556	49.8 ± 38.6 42.5 (3.2–161.3) n = 59	0.012	49.7 ± 37.2 42.5 (3.2–143.3) n = 35	50.0 ± 41.3 44.9 (3.5–161.3) n = 24
Postoperative dialysis	​	​	​	<0.0001	​	​
No postoperative dialysis	490 (78.4%)	458 (81.8%)	32 (49.2%)	​	20 (54.1%)	12 (42.9%)
Postoperative dialysis	135 (21.6%)	102 (18.2%)	33 (50.8%)	​	17 (45.9%)	16 (57.1%)
​	21	20	1	​	1	0
Postoperative sepsis	​	​	​	<0.0001	​	​
No	562 (89.6%)	518 (91.5%)	44 (72.1%)	​	26 (78.8%)	18 (64.3%)
Yes	65 (10.4%)	48 (8.5%)	17 (27.9%)	​	7 (21.2%)	10 (35.7%)
Missing	19	14	5	​	5	0

Data are presented as mean ± standard deviation, median (range) and number of observations, or number (percentage).

For test between two groups with respect to dichotomous variables Fisher’s exact test was used, for non-ordered categorical.

variables Chi-square test, and for continuous variables Mann-Whitney U-test.

PGD, primary graft dysfunction; LV, left ventricle; RV, right ventricle; MCS, mechanical circulatory support; ICU, intensive care unit.

### Association between PGD and mortality or re-transplantation

**FIGURE 2:**
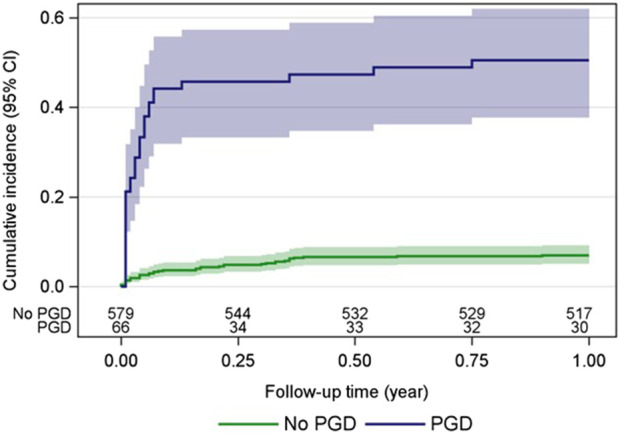
Cumulative incidence for time to all-cause mortality or re-Tx during first year by PGD.

The 30-day mortality or re-transplant rate in the PGD group was 45.5% compared with 3.3% in the non-PGD group (p < 0.0001). Death during hospitalization was 47% in the PGD group and 4.1% in the non-PGD group (p < 0.0001). 1-year mortality or re-HTx was 51.5% in the PGD group and 7.6% in the non-PGD group (Supplementary Table SA1; Figure 1). The median survival for the PGD group was less than 1 year compared to more than 15 years for the non-PGD group (Figure 2).

### Association between pre-HTx or intraoperative variables and PGD

Predictors of PGD in the age- and sex-adjusted logistic regression model were: age (adjusted (a)OR 0.80 (CI 0.66–0.96), p = 0.017; weight (aOR 0.90 (CI 0.82–0.98), p = 0.021); inverse height risk association (aOR 0.73 (CI 0.62–0.87), p = 0.0005); diagnosis (aOR 0.39 (CI 0.19–0.77), p = 0.0069); pre-HTx dialysis (aOR 7.03 (CI 1.79–27.5), p = 0.0051); pre-HTx sternotomy (aOR 2.51 (CI 1.46–4.29), p = 0.0008); ECC (aOR 1.75 (CI 1.45–2.11), p < 0.0001); arrhythmias pre-HTx (aOR 1.97 (CI 1.16–3.37), p = 0.013); and pre-HTx VAD (aOR 1.85 (CI 1.03–3.33), p = 0.039).

Independent predictors for PGD by multivariable logistic regression were: pre-HTx dialysis (aOR 6.73 (CI 1.46–31.6), p = 0.015); arrhythmias pre-HTx (aOR 2.08 (CI 1.16–3.75), p = 0.014); inverse height risk association (aOR 0.78 (CI 0.64–0.95), p = 0.012); longer ECC time (aOR 1.75 (CI 1.44–2.14, p < 0.0001), per 60 min increase) (Supplementary Table SA2).

### Association between pre-HTx or intraoperative variables and death of any cause

A stepwise Cox regression analysis of 1-year outcomes of death from any cause and re-HTx (Supplementary Table SA3) identified 11 predictors in the univariable analysis, and three were independently significant after adjustment for age and sex in the multivariable analysis. In the multivariable analysis, PGD had an almost 7-fold higher risk of death vs. non-PGD (aHR of 6.89 [CI 4.01–11.83], p < 0.0001, Supplementary Table SA3). Individuals transplanted 2011–2022 had a lower risk of death or re-HTx compared to 1984–1999, (aHR 0.27 [CI 0.14–0.52], p = 0.0001). For every 60 min of increased ECC time, the risk of death or re-HTx was increased (aHR 1.38 [CI 1.17–1.63], p = 0.0002).

## Discussion

Primary graft dysfunction occurred in 10.5% of all adult patients that underwent a HTx in our single center analysis. PGD was associated with a severely increased risk of early mortality with 50% of the patients not surviving the first year.

The incidence of PGD in the analysis was lower compared to other publications. Buchan conducted a meta-analysis in 2021 that included 36 studies. The pooled incidence of PGD was 20.5% [7]. Singh et al published a national study from the UK in 2019, 450 heart transplants were included and the incidence of moderate or severe PGD was 36% [13]. Dronovalli reported an incidence of 32.4%, however not based on the ISHLT criteria, based on the 290 transplantations in a single center study [14]. Based on ISHLT criteria and a data set from Duke University Hospital between 2009 and 2014, Nicoara et al reported a PGD incidence of 31% in 317 patients [15]. However, Rhee et al included 570 patients an analysis and reported an PGD incidence of 6.1%, comparable to our study also based on ISHLT criteria [16]. D’Alessandro et al conducted a study including 1221 donor hearts in-between 2015–2022, and based on ISHLT criteria the overall incidence for PGD was 18.5% [17]. Similar to our data set, the studies above included a majority of male recipients, with the mean age under 60 years, and similar preoperative conditions.

Despite the lower incidence of PGD in our population, both 30-day (45.5%) and 1-year mortality or re-Tx (51.5%) in the PGD population were higher compared to the other above-mentioned studies with 30-day mortality being 14.5%–39.3% [13–16] and 1-year mortality of 27.5%–41.5% [14, 16]. In our population the majority of the PGD cases were either severe PGD-LV or PGD-RV and these more severe forms carry a higher risk of mortality. This should be compared to the before mentioned studies where a large part of the cohort consisted of the mild or moderate PGD-LV. In Rhee’s study population, only 0.5% of subjects were diagnosed with PGD-RV, and of the 6.1% with PGD-LV, 40% had the moderate and 57.1% had the severe form [16]. In the meta-analysis, the pooled incidence of PGD-LV was mild 3.5%, moderate 6.6% and severe PGD-LV 7.7%. The incidence of PGD-RV was only 1.6% [7]. In D'Alessandro’s study the incidence of severe PGD was 8.2%. The 30-day and 1 year mortality in the severe PGD group was 19% respectively 31.2%. Their multivariable analysis showed that the recipients who develop severe PGD face a 7.8-fold higher risk of death within 1 year compared to those who do not. These results are similar to our study with a HR of 6.89 in the PGD-group (Supplementary Table SA3) [17]. The overall lower mortality rate can therefore be explained by two primarily factors. Firstly, a larger portion of the PGD cohorts consisting of the mild or moderate form and secondly, our patient inclusion spanned a broader temporal range during which therapeutic modalities have undergone significant advancements and refinements.

Our study also aimed to identify donor, pre- and intraoperative variables associated with an increased risk of developing PGD after heart transplant. Most interestingly, no donor factor was associated with PGD, instead recipient factors linked to a higher degree of disease (dialysis, arrhythmias) or more complex surgery (long ECC-time) were the ones found significant in multivariable analyses. Although donor data was not detailed, this indicated that recipient factors may be of higher importance for development of PGD. The significance of recipient length may be attributed to challenges in finding an appropriate size match of an appropriate donor heart, resulting in prolonged waiting times for transplantation and potentially under sizing of donors in relationship to the recipient. Singh and Buchan et al both reported that under sized donor hearts, as measured by predicted heart mass, were associated with the development of PGD postoperatively. In a similar study by Singh et al, they identified additional risk factors, such as elevated recipient body mass index, preoperative creatinine, recipient hospitalized at the time of transplantation, ischemic cardiomyopathy, undersized donor (by difference in predicted heart mass of greater than 30%), and requirement for ECMO prior to transplantation. The pre-operative recipient related risk-factors in Buchans meta-analysis was usage of amiodarone pre-Tx, an increase in creatinine levels, pre-Tx VAD or ECMO, where the results certainty regarding pre-tx VAD or ECMO was deemed moderate due to high inconsistency in-between the studies. We found that pre-Tx VAD was associated with PGD in an age- and sex-adjusted analyses. However, in our analysis neither ECMO nor pre-Tx VAD were related to increased risk of PGD in the multivariable analyses, in the presence of other strong association variables, most likely related to a type-2-error, indicating a small cohort. Nonetheless, decreased kidney function was in both our, Buchans and Singh et al’s studies significantly related to increased risk of PGD [7, 13].

Part of the criteria for determining PGD is the inotropic score. To differentiate between mild and moderate PGD, a threshold of 10 is required. The fact that we did not diagnose anyone in our population with mild PGD-LV may be since this threshold does not adequately reflect the clinical reality observed in post-transplant care. In practice, early and aggressive use of inotropic support within the first 24 h post-transplantation appears to be common, resulting in most patients exhibiting inotropic scores above 10, regardless of their PGD status. This trend is evident in the data presented in Table 4, where even non-PGD patients demonstrate a mean inotropic score of 29, well above the current threshold for moderate PG. The matter was also discussed by Kaye et al, suggesting removing norepinephrine all together from the score to prevent up grading patients from mild to moderate PGD, something that needs to be discussed further [18].

### Limitations and strengths

The limitations include the retrospective nature, some missing data and limited information from echocardiography that was simply not used routinely in the early eras of HTx. Furthermore, in the current study moderate PGD were diagnosed primarily based on hemodynamic criteria as echocardiography within 24 h of transplantation was usually not available. Thus, the incidence of moderate PGD could be underestimated leading to a PGD population consisting of more severe forms of PGD which in turn may have overestimated the mortality [14]. Furthermore, the lack of relevant donor data prevents us from investigating mismatch issues. In addition, the study spans many decades without detailing such things as preservation techniques and perioperative management. Finally the study has been worked up over several years but does not take into account the new and modified PGD definition.

However, the strength of this study is the consecutively transplanted and followed population, with all charts manually checked for PGD retrospectively, in a system where all patients can be tracked according to social security numbers. There are therefore no patients lost to follow-up.

In conclusion, primary graft dysfunction, using the ISHLT 2014 definitions, was a frequent and severe complication following HTx, characterized by poor prognosis in terms of both 30-day and 1-year survival rates. Four independent predictors for PGD were pre-HTx dialysis, pre-transplant arrythmias, inverse height association and longer ECC time. However, there were no donor variables identified as predictors for PGD. However, caution in interpretations is encouraged since cohort is relatively small.

## Data Availability

The original contributions presented in the study are included in the article/Supplementary Material, further inquiries can be directed to the corresponding author.
