# Supplementary material for: Incidence, predictors and outcomes following primary graft dysfunction after cardiac transplantation
Source: Transpl Int. 2026 Jun 30;39:16083. doi: 10.3389/ti.2026.16083 (PMC13367075; doi:10.3389/ti.2026.16083)
Supplement: Supplementary file 1 [file DataSheet1.pdf]

Appendix Table 1.

Patient outcome data

Variable	Total N=646	No PGD N=580	PGD N=66	PGD-LV N=38	PGD-RV N=28	No postoperative MCS N=15	Postoperative MCS N=50
In-hospital death							
No	591 (91.5%)	556 (95.9%)	35 (53.0%)	15 (39.5%)	20 (71.4%)	14 (93.3%)	21 (42.0%)
Yes	55 (8.5%)	24 (4.1%)	31 (47.0%)	23 (60.5%)	8 (28.6%)	1 (6.7%)	29 (58.0%)
All-cause mortality during 30 days							
No	598 (92.6%)	561 (96.7%)	37 (56.1%)	17 (44.7%)	20 (71.4%)	14 (93.3%)	23 (46.0%)
Yes	48 (7.4%)	19 (3.3%)	29 (43.9%)	21 (55.3%)	8 (28.6%)	1 (6.7%)	27 (54.0%)
All-cause mortality or re-Tx during 30 days							
No	597 (92.4%)	561 (96.7%)	36 (54.5%)	16 (42.1%)	20 (71.4%)	14 (93.3%)	22 (44.0%)
Yes	49 (7.6%)	19 (3.3%)	30 (45.5%)	22 (57.9%)	8 (28.6%)	1 (6.7%)	28 (56.0%)
All-cause mortality during 1 year							
No	573 (88.7%)	540 (93.1%)	33 (50.0%)	15 (39.5%)	18 (64.3%)	14 (93.3%)	19 (38.0%)
Yes	73 (11.3%)	40 (6.9%)	33 (50.0%)	23 (60.5%)	10 (35.7%)	1 (6.7%)	31 (62.0%)
All-cause mortality or re-Tx during 1 year							
No	568 (87.9%)	536 (92.4%)	32 (48.5%)	14 (36.8%)	18 (64.3%)	14 (93.3%)	18 (36.0%)
Yes	78 (12.1%)	44 (7.6%)	34 (51.5%)	24 (63.2%)	10 (35.7%)	1 (6.7%)	32 (64.0%)
All-cause mortality during follow-up							
No	417 (64.6%)	392 (67.6%)	25 (37.9%)	12 (31.6%)	13 (46.4%)	12 (80.0%)	13 (26.0%)
Yes	229 (35.4%)	188 (32.4%)	41 (62.1%)	26 (68.4%)	15 (53.6%)	3 (20.0%)	37 (74.0%)
All-cause mortality or re-Tx during follow-up							
No	401 (62.1%)	378 (65.2%)	23 (34.8%)	10 (26.3%)	13 (46.4%)	11 (73.3%)	12 (24.0%)
Yes	245 (37.9%)	202 (34.8%)	43 (65.2%)	28 (73.7%)	15 (53.6%)	4 (26.7%)	38 (76.0%)
Data are presented as number (percentage).							

PGD= primary graft dysfunction. LV=left ventricle. RV= right ventricle. MCS= mechanical circulatory support. re-Tx= Re-transplantation.

Appendix Table 2.

Association between pre-Tx and intraoperative variables and PGD using adjusted logistic regression

				Age- and sex adjusted models		Multivariable model*
**Variable**	**Values**	**Missing data**	**Number of PGDs per value**	**Adjusted OR (95% CI)**	**AUC**	**Adjusted OR (95% CI)**
Sex (ref Male)	Male	0	49 (10.0%)			
	Female	0	17 (10.8%)	1.01 (0.56 - 1.83) p=0.96	0.57	0.53 (0.23 - 1.22) p=0.14
Age (per 10 years increase)	<=median	0	36 (11.1%)			
	>median	0	30 (9.3%)	0.80 (0.66 - 0.96) p=0.017	0.57	0.83 (0.67 - 1.02) p=0.08
Age category (ref <30y)	<30y	0	14 (20.0%)			
	30-<40y	0	6 (8.0%)	0.41 (0.10 - 1.78) p=0.24	0.62	
	40-<50y	0	12 (9.2%)	0.56 (0.07 - 4.61) p=0.59		
	50-<60y	0	27 (11.7%)	0.85 (0.05 - 15.32) p=0.91		
	60+y	0	7 (5.0%)	0.38 (0.01 - 15.00) p=0.61		
Weight (per 5 kg increase)	<=median	5	45 (14.0%)			
	>median	5	20 (6.3%)	0.90 (0.82 - 0.98) p=0.021	0.62	
Height (per 5 cm increase)	<=median	5	45 (13.2%)			
	>median	5	20 (6.7%)	0.73 (0.62 - 0.87) p=0.0005	0.65	0.78 (0.64 - 0.95) p=0.012
BMI (per 1 kg/m^2 increase)	<=median	6	38 (11.7%)			
	>median	6	27 (8.6%)	0.97 (0.91 - 1.04) p=0.41	0.58	
Diagnosis (ref IHD)	IHD	21	20 (12.7%)			
	DCM	21	23 (6.9%)	0.39 (0.19 - 0.77) p=0.0065	0.65	
	Other	21	21 (15.8%)	0.87 (0.41 - 1.84) p=0.72		
Smoking (ref No)	No	16	39 (11.2%)			
	Smoked within 6 months	16	4 (7.8%)	0.67 (0.23 - 1.99) p=0.47	0.58	
	Previous smoker (>6 months)	16	20 (8.7%)	0.89 (0.49 - 1.61) p=0.69		
Stroke/TIA (ref No)	No	12	51 (9.2%)			
	Yes	12	12 (15.2%)	1.75 (0.88 - 3.46) p=0.11	0.59	
Peripheral vascular disease (ref No)	No	15	59 (9.7%)			
	Yes	15	5 (20.8%)	2.93 (1.04 - 8.29) p=0.043	0.59	
Dialysis (ref No)	No	22	57 (9.3%)			
	Yes	22	4 (44.4%)	7.03 (1.79 - 27.53) p=0.0051	0.59	6.73 (1.46 - 31.06) p=0.015
Previous sternotomy	No	9	27 (6.8%)			

	Yes	9	37 (15.4%)	2.51 (1.46 - 4.29) p=0.0008	0.65	
Codarone (ref No)	No	20	44 (8.9%)			
	Yes	20	17 (12.9%)	1.61 (0.88 - 2.93) p=0.12	0.58	
Inotrope (ref No)	No	28	41 (8.8%)			
	Yes	28	19 (12.3%)	1.36 (0.75 - 2.44) p=0.31	0.56	
Levosimendan (ref No)	No	13	49 (10.4%)			
	Levosimendan within 6 months	13	6 (6.6%)	0.64 (0.26 - 1.53) p=0.31	0.55	
	Levosimendan continuously up to Tx	13	7 (9.7%)	0.97 (0.42 - 2.24) p=0.94		
NYHA (ref I-II)	I-II	30	1 (3.2%)			
	III	30	42 (10.0%)	3.39 (0.45 - 25.63) p=0.24	0.58	
	IV	30	17 (10.4%)	3.16 (0.40 - 24.77) p=0.27		
Arrhythmias (ref No)	No	19	32 (7.8%)			
	Yes	19	30 (13.8%)	1.97 (1.16 - 3.37) p=0.013	0.61	2.08 (1.16 - 3.75) p=0.014
VAD (ref No)	No	14	43 (8.5%)			
	Yes	14	20 (15.6%)	1.85 (1.03 - 3.33) p=0.039	0.60	
PCWP (per 1 unit increase)	<=median	48	29 (9.4%)			
	>median	48	28 (9.7%)	1.01 (0.97 - 1.04) p=0.71	0.51	
CVP (per 1 unit increase)	<=median	152	19 (7.3%)			
	>median	152	25 (10.7%)	1.03 (0.98 - 1.08) p=0.28	0.57	
Calendar year (ref 1984-1999)	1984-1999	0	21 (10.8%)			
	2000-2010	0	27 (15.8%)	1.60 (0.86 - 2.96) p=0.13	0.64	
	2011-2022	0	18 (6.4%)	0.62 (0.32 - 1.20) p=0.15		
Donor sex (ref Male)	Male	0	40 (9.6%)			
	Female	0	26 (11.3%)	1.14 (0.65 - 2.00) p=0.64	0.58	
Donor age (per 10 years increase)	<=median	1	31 (9.4%)			
	>median	1	35 (11.1%)	1.12 (0.94 - 1.35) p=0.21	0.59	
Donor blood group (ref 0)	0	3	30 (10.8%)			
	A	3	28 (9.8%)	0.93 (0.54 - 1.62) p=0.81	0.57	
	B	3	7 (9.7%)	0.94 (0.39 - 2.25) p=0.89		
	AB	3	1 (11.1%)	1.21 (0.14 - 10.20) p=0.86		
Donor height (per 5 cm increase)	<=median	5	33 (10.2%)			
	>median	5	33 (10.4%)	0.98 (0.87 - 1.11) p=0.78	0.58	
Donor weight (per 5 kg increase)	<=median	3	33 (10.2%)			
	>median	3	33 (10.3%)	1.06 (0.99 - 1.14) p=0.12	0.60	
Reason for death (ref ICB)	Unknown	87	0 (0.0%)	0.00 (0.00 - I) p=0.98	0.60	

	ICB	87	31 (11.5%)			
	Trauma	87	16 (10.5%)	0.85 (0.45 - 1.62) p=0.62		
	Thromboembolic	87	1 (6.3%)	0.46 (0.06 - 3.63) p=0.46		
	Other	87	7 (7.3%)	0.59 (0.25 - 1.39) p=0.23		
Ischemic time (per 60min increase)	<=median	22	27 (8.7%)			
	>median	22	37 (11.9%)	1.27 (0.98 - 1.65) p=0.07	0.61	
ECC (per 60min increase)	<=median	14	18 (5.7%)			
	>median	14	47 (15.0%)	1.75 (1.45 - 2.11) p=<.0001	0.72	1.75 (1.44 - 2.14) p=<.0001
Aortaocklusion (per 60min increase)	<=median	181	23 (9.9%)			
	>median	181	26 (11.2%)	1.07 (0.79 - 1.44) p=0.67	0.62	
* AUC for the multivariable model was 0.76.						

BMI= Body mass index. NYHA= New York Heart Association. TIA= Transitory ischemic attack. PCWP= pulmonary capillary wedge pressure. CVP= Central venous pressure. VAD= Ventricular assist device. ECC= Extra corporeal circulation.

Appendix Table 3
Association between pre-Tx and intraoperative variables and time to all-cause death or re-Tx during first year using adjusted Cox regression

Variable	Value	n(%) events	Crude event date (95% CI)	Adjusted model Hazard Ratio (95% CI)*	Multivariable model Hazard Ratio (95% CI)
PGD	No	44 (7.6%)	8.2 (6.0-11.0)		
	Yes	34 (51.5%)	101.3 (70.2-141.6)	10.01 (6.28 - 15.94) p=<.0001	6.89 (4.01 - 11.83) p=<.0001
Sex	Male	66 (13.5%)	15.6 (12.1-19.9)		
	Female	12 (7.6%)	8.1 (4.2-14.2)	0.52 (0.28 - 0.96) p=0.038	0.53 (0.27 - 1.05) p=0.07
Age (per 10 years increase)	<=median (5.3)	45 (13.8%)	15.9 (11.6-21.3)		
	>median (5.3)	31 (9.7%)	10.8 (7.3-15.3)	0.80 (0.68 - 0.95) p=0.0092	0.95 (0.80 - 1.13) p=0.57
Age category	<30y	13 (18.6%)	23.1 (12.3-39.5)		
	30-<40y	10 (13.3%)	15.5 (7.4-28.5)	0.73 (0.21 - 2.56) p=0.63	
	40-<50y	15 (11.5%)	12.7 (7.1-21.0)	0.67 (0.10 - 4.43) p=0.68	
	50-<60y	32 (13.9%)	16.0 (10.9-22.6)	0.80 (0.06 - 10.63) p=0.87	
	60+y	8 (5.8%)	6.1 (2.6-12.0)	0.35 (0.01 - 9.31) p=0.53	
Weight (per 5 kg increase)	<=median (15.8)	42 (13.0%)	14.8 (10.7-20.0)		
	>median (15.8)	30 (9.5%)	10.5 (7.1-15.0)	0.92 (0.85 - 1.00) p=0.051	
Height (per 5 cm increase)	<=median (35.2)	37 (10.9%)	12.2 (8.6-16.8)		
	>median (35.2)	35 (11.7%)	13.2 (9.2-18.3)	0.82 (0.70 - 0.95) p=0.0092	
BMI (per 1 kg/m^2 increase)	<=median (25.2)	43 (13.5%)	15.4 (11.2-20.8)		
	>median (25.2)	28 (8.8%)	9.7 (6.4-14.0)	0.96 (0.91 - 1.02) p=0.23	
Diagnosis	IHD	24 (15.2%)	17.7 (11.3-26.3)		
	DCM	33 (9.9%)	11.0 (7.5-15.4)	0.57 (0.32 - 1.02) p=0.06	
	Other	19 (14.3%)	16.7 (10.0-26.0)	0.80 (0.41 - 1.57) p=0.52	
Smoking	No	36 (10.3%)	11.6 (8.1-16.1)		
	Smoked within 6 months	5 (9.8%)	10.6 (3.4-24.7)	1.07 (0.42 - 2.73) p=0.89	
	Previous smoker (>6 months)	31 (13.4%)	15.4 (10.4-21.8)	1.67 (0.99 - 2.79) p=0.053	
Stroke/TIA	No	65 (11.7%)	13.3 (10.2-16.9)		
	Yes	7 (8.9%)	9.6 (3.9-19.8)	0.72 (0.33 - 1.57) p=0.40	
Peripheral vascular disease	No	67 (11.0%)	12.4 (9.6-15.7)		
	Yes	5 (20.8%)	26.9 (8.7-62.7)	2.28 (0.91 - 5.76) p=0.08	
Dialysis	No	68 (11.1%)	12.4 (9.6-15.7)		
	Yes	4 (44.4%)	77.7 (21.2-198.9)	5.99 (2.13 - 16.83) p=0.0007	
Previous sternotomy	No	35 (8.8%)	9.6 (6.7-13.4)		

	**Yes**	39 (16.3%)	19.4 (13.8-26.5)	1.77 (1.11 - 2.84) p=0.017	
**Codarone**	**No**	49 (9.9%)	11.0 (8.1-14.5)		
	**Yes**	22 (16.7%)	19.9 (12.5-30.2)	1.98 (1.19 - 3.30) p=0.0085	
**Inotrope**	**No**	51 (11.0%)	12.3 (9.2-16.2)		
	**Yes**	20 (13.0%)	14.8 (9.1-22.9)	1.19 (0.70 - 2.01) p=0.52	
**Levosimendan**	**No**	67 (14.3%)	16.4 (12.7-20.8)		
	**Levosimendan within 6 months**	7 (7.7%)	8.6 (3.4-17.7)	0.58 (0.27 - 1.28) p=0.18	
	**Levosimendan continuously up to Tx**	1 (1.4%)	1.5 (0.0-8.1)	0.10 (0.01 - 0.73) p=0.023	
**NYHA**	**I-II**	1 (3.2%)	3.6 (0.1-20.3)		
	**III**	49 (11.6%)	13.1 (9.7-17.3)	3.85 (0.53 - 27.93) p=0.18	
	**IV**	18 (11.0%)	12.3 (7.3-19.4)	3.32 (0.44 - 24.96) p=0.24	
**Arrhythmias**	**No**	39 (9.5%)	10.6 (7.5-14.4)		
	**Yes**	33 (15.1%)	17.7 (12.2-24.8)	1.90 (1.18 - 3.05) p=0.0078	
**VAD**	**No**	58 (11.5%)	12.9 (9.8-16.7)		
	**Yes**	16 (12.5%)	14.4 (8.3-23.5)	0.98 (0.56 - 1.72) p=0.95	
**PCWP (per 1 unit increase)**	**<=median (20.0)**	35 (11.3%)	12.8 (8.9-17.8)		
	**>median (20.0)**	33 (11.5%)	12.9 (8.9-18.1)	1.02 (0.98 - 1.05) p=0.33	
**CVP (per 1 unit increase)**	**<=median (9.0)**	27 (10.4%)	11.6 (7.6-16.8)		
	**>median (9.0)**	33 (14.1%)	16.2 (11.1-22.7)	1.02 (0.99 - 1.06) p=0.23	
**Calendar year**	**1984-1999**	38 (19.6%)	23.4 (16.6-32.2)		
	**2000-2010**	25 (14.6%)	16.9 (10.9-24.9)	0.80 (0.48 - 1.32) p=0.38	0.60 (0.35 - 1.02) p=0.06
	**2011-2022**	15 (5.3%)	5.8 (3.2-9.5)	0.28 (0.15 - 0.51) p=<.0001	0.27 (0.14 - 0.52) p=0.0001
**Donor sex**	**Male**	50 (12.0%)	13.6 (10.1-18.0)		
	**Female**	28 (12.2%)	13.8 (9.2-19.9)	1.22 (0.75 - 1.99) p=0.42	
**Donor age (per 10 years increase)**	**<=median (4.3)**	37 (11.2%)	12.6 (8.9-17.4)		
	**>median (4.3)**	38 (12.1%)	13.7 (9.7-18.8)	1.11 (0.94 - 1.30) p=0.21	
**Donor blood group**	**0**	38 (13.7%)	15.9 (11.3-21.9)		
	**A**	30 (10.5%)	11.6 (7.8-16.5)	0.78 (0.48 - 1.26) p=0.31	
	**B**	8 (11.1%)	12.8 (5.5-25.2)	0.73 (0.33 - 1.65) p=0.46	
	**AB**	0 (0.0%)	0.0 (-41.0)	0.00 (0.00 -) p=0.98	
**Donor height (per 5 cm increase)**	**<=median (35.2)**	35 (10.8%)	12.1 (8.4-16.9)		
	**>median (35.2)**	39 (12.3%)	14.0 (10.0-19.2)	1.02 (0.91 - 1.15) p=0.71	
**Donor weight (per 5 kg increase)**	**<=median (15.2)**	37 (11.5%)	13.0 (9.1-17.9)		
	**>median (15.2)**	38 (11.9%)	13.4 (9.5-18.4)	0.97 (0.90 - 1.04) p=0.39	
**Reason for death**	**Unknown**	1 (4.0%)	4.3 (0.1-24.2)		

	**ICB**	36 (13.3%)	15.1 (10.6-20.9)	3.00 (0.41 - 21.97) p=0.28	
	**Trauma**	25 (16.4%)	19.3 (12.5-28.4)	3.65 (0.49 - 27.00) p=0.21	
	**Thromboembolic**	2 (12.5%)	14.3 (1.7-51.5)	2.70 (0.24 - 29.90) p=0.42	
	**Other**	3 (3.1%)	3.2 (0.7-9.4)	0.68 (0.07 - 6.51) p=0.73	
**Ischemic time (per 60min increase)**	**<=median (3.1)**	32 (10.2%)	11.3 (7.8-16.0)		
	**>median (3.1)**	38 (12.3%)	13.9 (9.9-19.1)	1.07 (0.84 - 1.35) p=0.58	
**ECC (per 60min increase)**	**<=median (2.5)**	31 (9.5%)	10.4 (7.1-14.7)		
	**>median (2.5)**	41 (13.6%)	15.7 (11.3-21.3)	1.65 (1.43 - 1.91) p=<.0001	1.38 (1.17 - 1.63) p=0.0002
**Cross clamp duration at implant (per 60min increase)**	**<=median (1.3)**	29 (12.8%)	14.5 (9.7-20.8)		
	**>median (1.3)**	26 (11.0%)	12.4 (8.1-18.1)	0.98 (0.74 - 1.28) p=0.86	
* Cox regression adjusted for age and sex.					

PGD= Primary graft dysfunction. BMI= Body mass index. NYHA= New York Heart Association. TIA= Transitory ischemic attack. PCWP= pulmonary capillary wedge pressure. CVP= Central venous pressure. VAD= Ventricular assist device. ECC= Extra corporeal circulation.

Appendix Figure 1

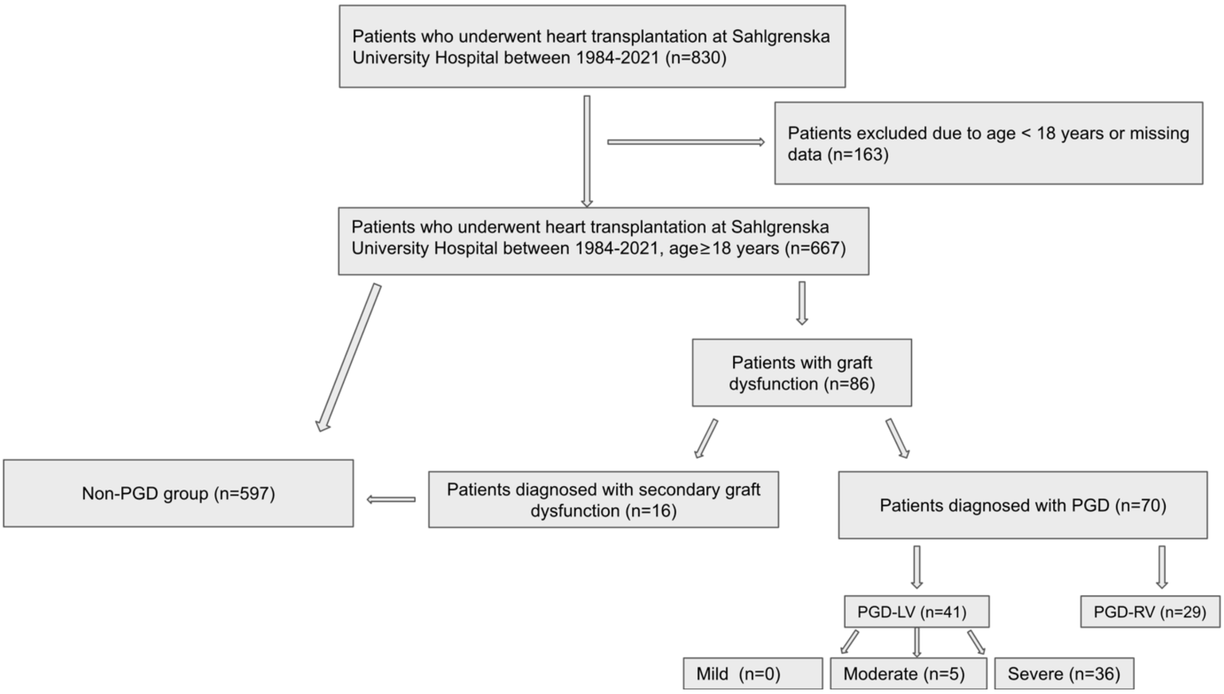

Flowchart of the inclusions of patients. PGD= primary graft dysfunction. LV=left ventricle. RV= right ventricle.

Appendix Figure 2

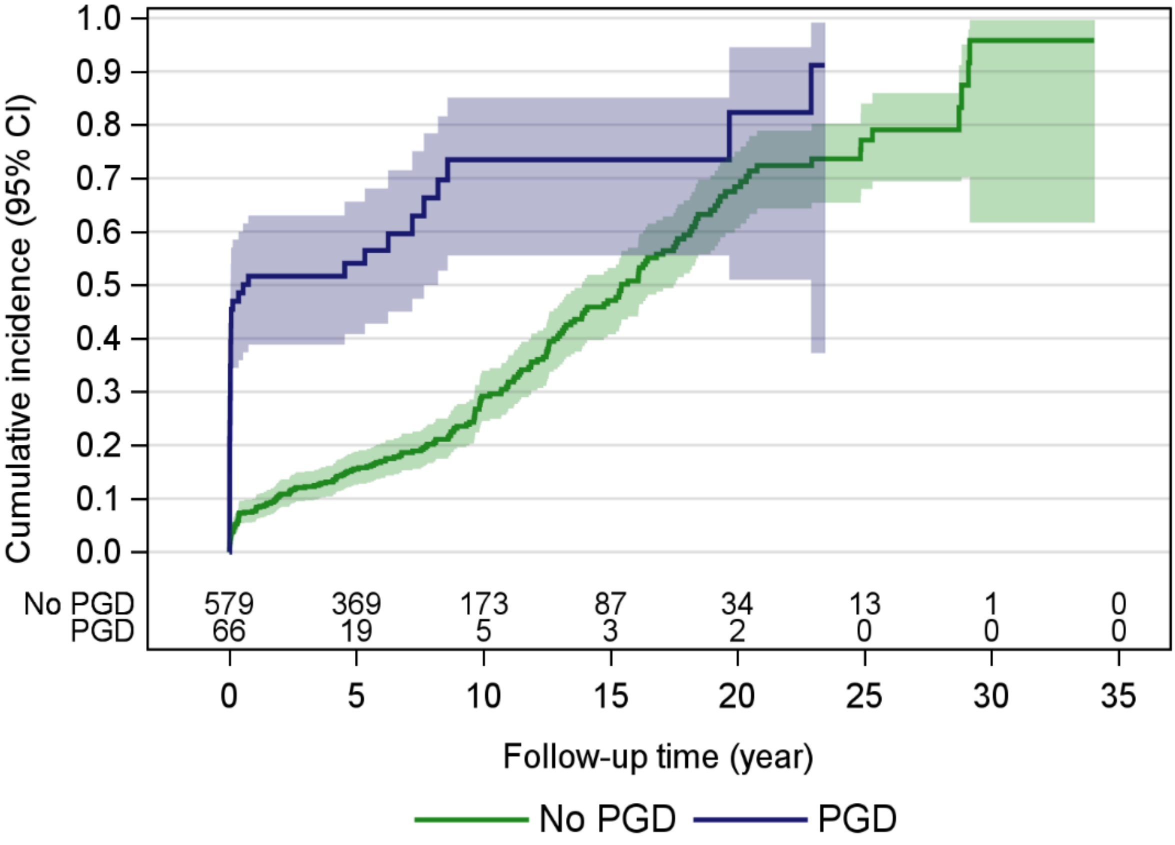

Cumulative incidence for time to all-cause mortality or re-Tx during whole follow-up period by PGD
